# Investigation of Lithium–Ion Battery Performance Utilizing Magnetic Controllable Superionic Conductor Li_3_(V_1–*x*_Fe_*x*_)_2_(PO_4_)_3_/C (*x* = 0.05 and 0.10)

**DOI:** 10.1021/acsomega.4c01757

**Published:** 2024-06-21

**Authors:** Yu-Ting Lee, Yi-Tsen Chen, Jun-Yi Cheng, Chun-Chuen Yang, Kuen-Song Lin

**Affiliations:** † Department of Physics, Chung Yuan Christian University, No. 200, Zhongbei Rd., Zhongli, Taoyuan 320314, Taiwan; ‡ Department of Photonics, National Cheng Kung University, No. 1, University Rd., Tainan 701401, Taiwan; § Department of Physics, National Central University, No. 300, Zhongda Rd., Zhongli, Taoyuan 320317, Taiwan; ∥ Department of Chemical Engineering and Material Science, Yuan Ze University, No. 135, Yuandong Rd., Zhongli, Taoyuan 320315, Taiwan

## Abstract

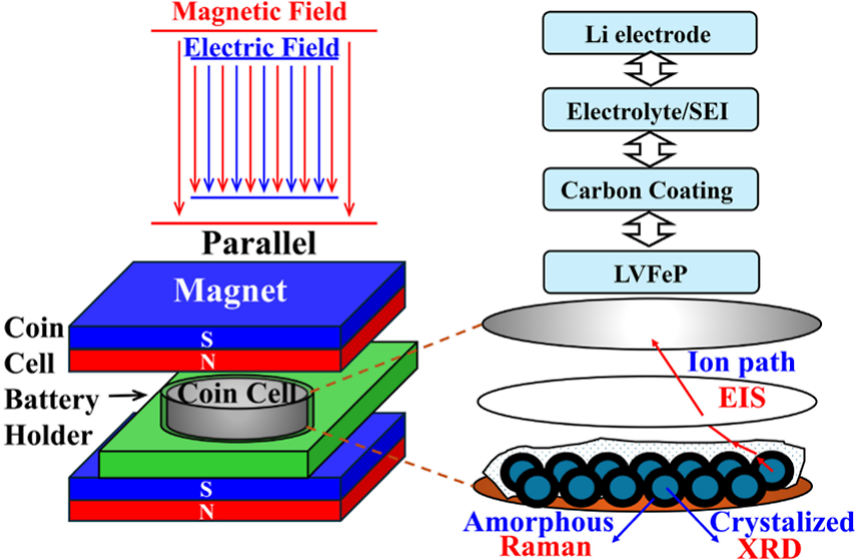

Lithium–ion batteries with Li_3_V_2_(PO_4_)_3_/C as the cathode have been a popular research topic in recent years; however, studies of the effects of external magnetic fields on them are less common. This study investigates the effects of an external magnetic field applied parallel to the direction of the anode and cathode on the ion transport through iron-doped Li_3_(V_1–*x*_Fe_*x*_)_2_(PO_4_)_3_, the outer carbon coating, the film/electrolyte/separator, and up to the lithium metal electrode on a microscopic level. The results reveal that for the *x* = 0.05 sample with lower doping, the magnetostriction expansion of Li_3_(V_1–*x*_Fe_*x*_)_2_(PO_4_)_3_ and the magnetostrictive contraction effect of the outer ordered carbon layer cancel each other out, resulting in no significant enhancement of the battery’s energy and power density due to the external magnetic field. In contrast, the *x* = 0.1 sample, lacking magnetostrictive contraction in the outer ordered carbon layer, shows that its energy and power density can be influenced by the magnetic field. Under zero magnetic field, the cyclic performance exhibits superior average capacity performance in the *x* = 0.05 sample, while the *x* = 0.1 sample shows a lower decay rate. Both samples are affected by the magnetic field; however, the *x* = 0.1 sample performs better under magnetic conditions. In particular, in the C-rate tests under a magnetic field, the sample with *x* = 0.1 showed a significant relative reduction in capacity decay rate by 20.18% compared to the sample with *x* = 0.05.

## 1 Introduction

The widespread use of portable devices and the development of electric vehicles have spurred the demand for portable power sources. Lithium–ion batteries, as energy storage devices with high storage capacity, low weight, and the ability to be cycled multiple times, have attracted the attention of material scientists. Li_3_V_2_(PO_4_)_3_ (abbreviated as LVP) is a superionic conductor that, due to its excellent ionic storage properties, is also used as an electrode material in ion batteries, gaining prominence and triggering extensive research since the early 2000s.^1−12^ However, LVP itself has poor conductivity. To enhance its performance, many studies have explored surface coating, with the most common method involving the use of citric acid in the process, which forms a carbon coating on the material surface,^13−15^ facilitating conductivity. Such carbon-coated LVP, typically denoted as LVP/C, improves performance when used as the cathode material in lithium batteries.^16−18^ For instance, Long Wang and colleagues reported that after 100 cycles at 1 C current (a discharge current that discharges the full capacity within 1 h), the capacity only dropped from 118 to 113 mA h/g.^19^ When the current density was increased, it was observed that LVP/C could still provide a capacity of 80 mA h/g under 24 C charging and discharging conditions. This demonstrates the advantages of using LVP/C as a cathode for lithium batteries, as it allows for multiple charging and discharging cycles with low capacity loss and can be charged and discharged at high current densities. In general usage scenarios, the application’s charge/discharge voltage platform is located at 4.07 V.^19^ LVP also exhibits an additional capacity corresponding to the V^3+^/V^2+^ redox reaction at 1.8 V.^20^ Besides carbon coating, there have been uses of copper coatings,^21^ multiple coating layers,^22^ or more complex configurations such as doping chlorine into already nitrogen-doped carbon layers.^23^ Additionally, altering the geometric morphology is a common method.^16−18^ To enhance the battery’s high charge/discharge performance, research on doping with other ions like Al^3+^,^24^ Sc^3+^,^25^ Ti^4+^,^26^ and Ru^4+^^23^ has been conducted. The synthesis of lithium–ion battery cathode material Li_3_(V_1–*x*_Fe_*x*_)_2_(PO_4_)_3_ (0 ≤ *x* ≤ 1) has been infrequently reported, primarily because it is challenging to coexist with low oxidation state V^3+^ and high oxidation state Fe^3+^ within the same Li_3_M_2_(PO_4_)_3_ framework using conventional one-step synthesis techniques. Knee et al. have reported the synthesis of Fe and Al-doped samples with *x* = 0.05 using a spray-drying-assisted carbothermal synthesis method.^20^ Changsong and colleagues have reported doping with Mg up to *x* = 0.13.^27^ Although Xudong and his team successfully achieved a full range substitution of V and Fe for 0 ≤ *x* ≤ 1 using a two-step synthesis method,^28^ in this study, using conventional processes, the upper limit for *x* was 0.1.

Previous research on applying magnetic fields to lithium–ion batteries has mainly focused on the effects of the magnetic field on electrochemical reactions,^29^ the influence of the magnetic field on the electrolyte for ion transport,^30^ the suppression of lithium dendrite growth using magnetic fields,^31^ or utilizing magnetic fields to rotate the orientation of graphite electrodes to reduce the Li^+^ ion pathway and enhance battery performance.^32^ However, studies on the magnetism of LVP or LVP/C and its impact on battery performance have been less frequently reported. Gavrilova and others have studied the magnetism of LVP/C, reporting paramagnetism above 50 K and, using ESR measurements after the first charge/discharge cycle at room temperature, that the vanadium ions could be fully reduced back to their initial valence state V^3+^.^33^ Moreover, the implications for battery performance were not further discussed. The magnetism of carbon surrounding the LVP layer has not been specifically reported. Generally, carbon exhibits diamagnetic behavior, but two-dimensional carbon, due to defects or oxidation, could develop other magnetic properties, including superparamagnetism or even ferromagnetism.^34^ Therefore, the carbon surrounding LVP may also exhibit varying magnetic characteristics due to defects or oxidation.

This study will discuss the effect of an external magnetic field on the ion propagation path (from LVFeP/C→ carbon layer → SEI film/electrolyte/separator → lithium metal) after doping the magnetic iron ion *x* = 0.05 and 0.10 into Li_3_(V_1–*x*_Fe_*x*_)_2_ (PO_4_)_3_/C (LVFeP/C) and the interaction between the ions and the crystal structure of the material and electrolyte. In our experimental design, we will specifically alternate using zero and specific external magnetic field strengths to repeatedly test the fabricated LVFeP materials. This method is aimed at determining whether the magnetically doped LVFeP materials are affected by magnetization, leading to permanent lattice expansion or contraction when the external magnetic field is applied and then removed. If permanent expansion or contraction occurs, this could potentially uncover a method for permanently altering ion channels, and subsequent tests would need to be conducted to determine how long this effect can be maintained over multiple charge–discharge cycles. Conversely, if no permanent expansion or contraction is observed, it will be necessary to maintain a constant external magnetic field in all subsequent tests to understand the effects of the magnetic field in opening or reducing the ion channels of LVFeP. On the other hand, the effects of magnetic fields on other lithium ions passing through the SEI film and electrolyte are analyzed through electrochemical impedance spectroscopy (EIS). By analyzing the changes in the physical properties of iron ions, the outer layer carbon film of LVFeP, the SEI film, and the electrolyte induced by the external magnetic field, we can understand the impact of the magnetic field on the overall performance of the battery from a microscopic perspective.

## 2 Experiments

The Li_3_(V_1–*x*_Fe_*x*_)_2_(PO_4_)_3_/C (*x* = 0.05, 0.1) produced by the chemical reaction method.

The formulas for preparing samples with *x* = 0.1 and 0.05 are shown in reaction schemes (1) or (2)
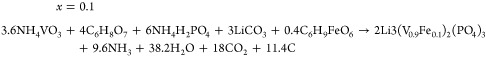
1
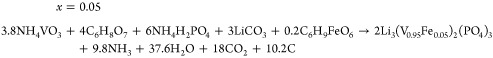
2Table 1 displays the names of the required chemicals, their CAS numbers, suppliers, product names, and purity levels.

**Table 1:** Chemicals Used in This Study, Including Chemical Names, CAS Numbers, Suppliers, Product Names and Specifications, and Purity Levels

chemical	CAS_number	supplier	product name	purity
NH_4_VO_3_	7803–55–6	Alfa Aesar	ammonium vanadium	99%
NH_4_H_2_PO_4_	7722–76–1	J. T. Baker	oxide,99% (metals basis)	≥98.0%
			ammonium phosphate monobasic	
C_6_H8O_7_·H_2_O	5949–29–1	J. T. Baker	citric acid monohydrate	99%
Li_2_CO_3_	554–13–2	J. T. Baker	lithium carbonate, 99%	99%
Fe(C_2_O_2_H_3_)_2_	3094–87–9	Alfa Aesar	iron acetate, anhydrous, Fe 29.5% min Power/Lump	Fe > 29.5%
Li	7439–93–2	UBIQ	lithium metal chips	99.95%
super P	1333–86–4	Timcal	super P conductive carbon black	>99%
PVDF	24937–79–9	Arkema	Kynar HSV 1800	
NMP, C_5_H_9_NO	872–50–4	Sigma-Aldrich	1-methyl-2-pyrrolidinone	99.5%
electrolyte		MSE Supplies	1 M LiPF_6_ in EC/DMC/DEC = 1:1:1 (v/v/v) electrolyte solution	
copper foil	7440–50–8	UBIQ	10 μm	>99.95%

The sample preparation process begins by dissolving ammonium metavanadate in DI water, followed by the addition of iron acetate according to the ratio specified in reaction 1 or 2 and stirring until uniform. Finally, citric acid is added and stirred until it is completely dissolved. Separately, ammonium dihydrogen phosphate is dissolved in 30 mL of DI water in a beaker, and the solution is slowly added dropwise into the former solution using a separating funnel. Lithium carbonate is dissolved in DI water using the same steps and slowly added dropwise to the solution. After the reagents were prepared, they were placed in an 80 °C oven to dry. Once dried, they are removed, ground, placed into aluminum crucibles, and then heated in a high-temperature furnace (Lindberg Blue/M 1500 °C STF55433C-1) at 350 °C for 4 h. After removal from the furnace, the desired samples are obtained.

The obtained samples were first identified for their purity and crystal phase by using a Bruker D8 ADVANCE Eco X-ray powder diffractometer. The K_α_ wavelength of the copper target was scanned from 9 to 60 degrees of the 2θ angle. The obtained data were fitted with the GSAS structure refinement software using the Rietveld method to calculate the crystal lattice structure.^35,36^

As the carbon layer in LVFeP/C is amorphous, a Horiba Lab Ram HR 520 Raman spectrometer was used to measure the vibrational spectra between atoms to analyze possible deformations in the channel. The experiment used a 632 nm red laser light source and was combined with a Linkam THMS600 temperature control kit to conduct temperature and magnetic field variation experiments from 80 to 300 K.

The fabrication of the cathode sheet for lithium batteries entails utilizing materials such as Li_3_(V_1–*x*_Fe_*x*_)_2_(PO_4_)_3_/C, Super-P (carbon powder), and poly(vinylidene fluoride) (PVDF) in an 8:1:1 ratio. These components are dissolved in *N*-methyl-2-pyrrolidone (NMP) to form a slurry. The slurry is meticulously mixed until homogeneous and then uniformly applied to a copper foil using a tabletop coater (Model CM-02VH, Shining Energy Co., Ltd.). A doctor blade setting of 75 μm is used to ensure consistent thickness. The coated foil is subsequently dried in an oven at 90 °C for 30 min and then cut into the required sizes for the cathode sheets.

For the electrolyte, a 1 M solution of LiPF_6_ is prepared by dissolving it in a mixed solvent comprising equal volumes of ethylene carbonate (EC) and dimethyl carbonate. The prepared cathode sheets are trimmed and placed in the bottom cap of a half-cell battery assembly within an argon-filled glovebox (MBraun LabMasterPro). This assembly process involves layering a separator, dispensing the LiPF_6_ electrolyte, and adding a lithium metal piece. A gasket and an upper cap are then placed, and the assemblies are pressed together to seal the half-cell battery. The complete assembly of the half-cell battery includes placing and securing the bottom cover, electrode, separator film, spacer, lithium metal, and top cover in a specified sequence.

The equipment used for the battery cycle charging and discharging tests was the Acutech systems BAT-750B. The experiment conducted charge and discharge tests within a voltage range of 0.02–3 V, measuring every 5 cycles of charging and discharging. A total of 45 cycles were performed at different rates, and capacity values were obtained under the condition of 0.1 C. The internal resistance and ion diffusion rate of the battery were measured using a Palmsens 4 electrochemical impedance spectrometer from the Palmsens company. The set current limits ranged from 100 pA to 100 mA; the applied AC voltage is 5 mV; and the frequency range was from 10 mHz to 100 Khz.

**Figure 1:**
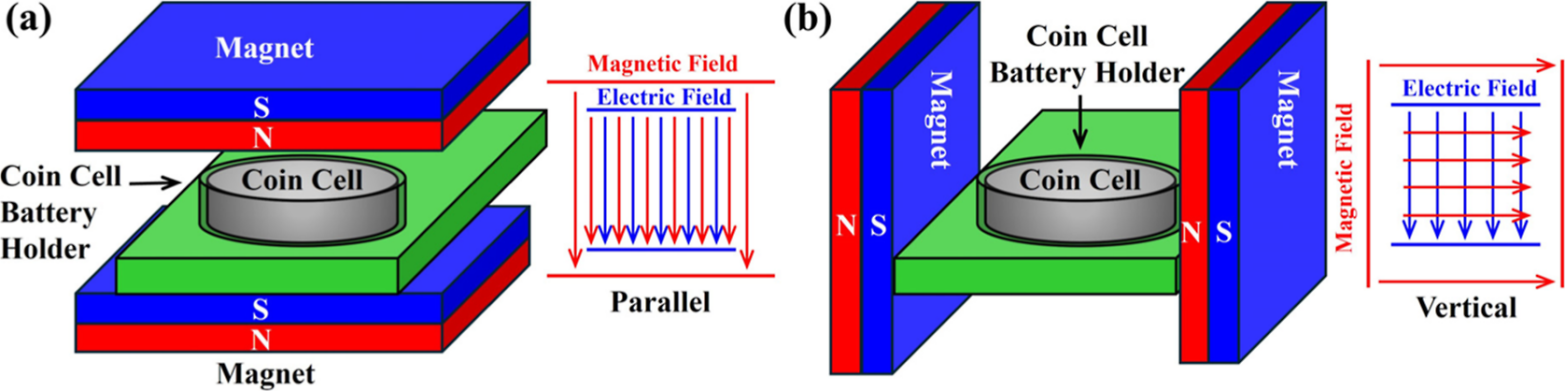
Setup methods for testing with externally applied magnetic fields in two orientations. (a) Magnetic field parallel to the direction of the positive and negative electrodes. (b) Magnetic field perpendicular to the direction of the positive and negative electrodes.

The relative positions of the battery in the external test magnetic field are two possibilities, as shown in Figure 1. Since the angle between the applied magnetic field and the electric field formed by the charging and discharging voltage is related to the direction of ion movement in the battery, careful planning of the experimental setup is crucial. The setup used in the charge–discharge tests in this study is illustrated in Figure 1a. Because the magnetic field is parallel to the electric field, the movement of lithium ions is only affected by the original electric field, and the effect caused by the magnetic field comes solely from the response of the sample’s own structure. However, if the setup is configured as shown in Figure 1b, the electric field and the magnetic field will form an interaction field, causing the magnetic field to deflect the direction of ion movement, resulting in an elongation of the migration path and potentially creating phenomena similar to the Hall effect. Consequently, there will be additional effects not originating from the material’s intrinsic response to the magnetic field. Since this study focuses only on the influence of the magnetic field on the structure of the sample itself, the setup depicted in Figure 1a is employed.

## 3 Results and Discussion

**Figure 2:**
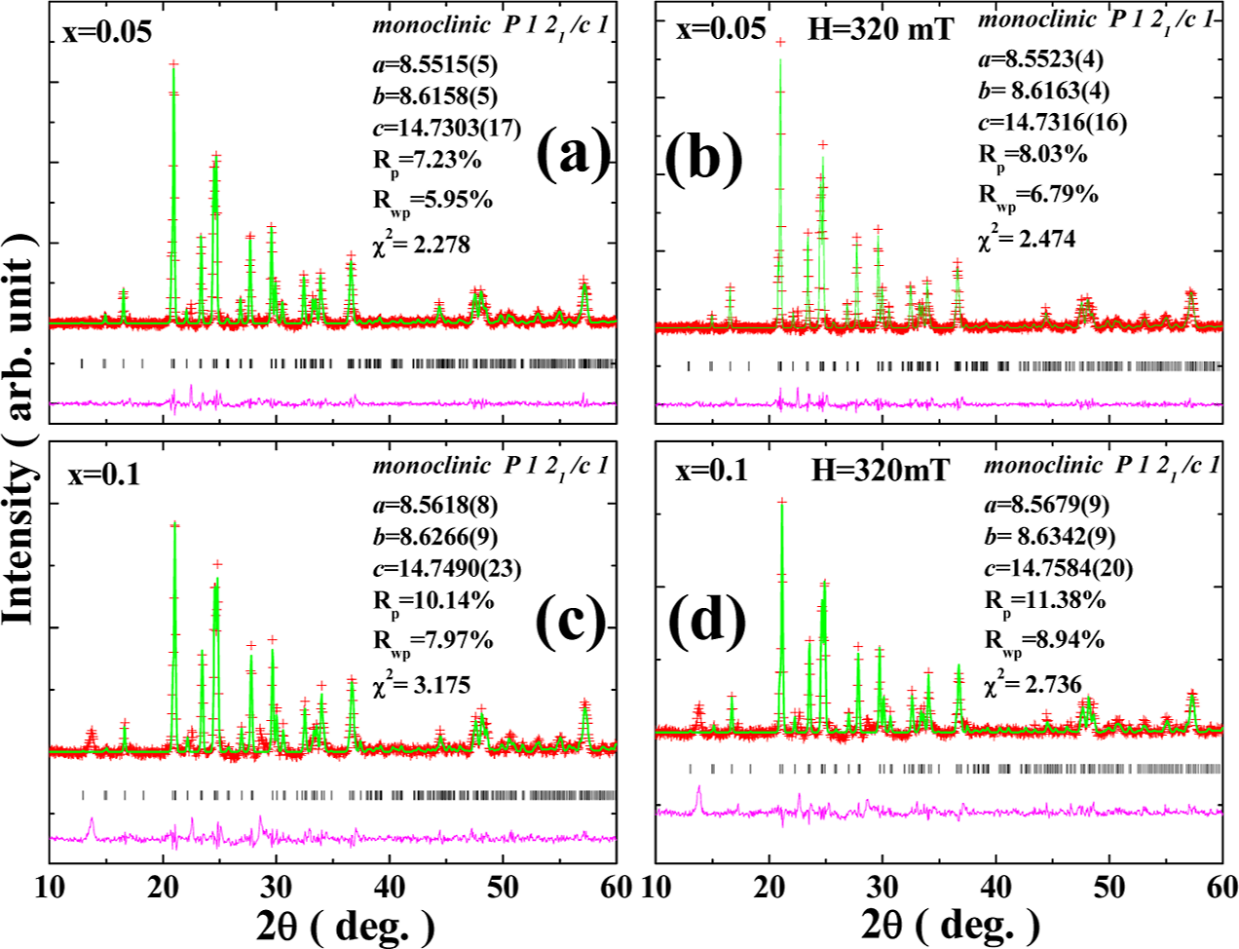
X-ray powder diffraction patterns for the *x* = 0.05 sample under (a) *H* = 0 mT, (b) *H* = 320 mT, and for the *x* = 0.1 sample under (c) *H* = 0 mT and (d) *H* = 320 mT.

Figure 2 shows the X-ray diffraction (XRD) powder diffraction patterns of LVFeP. Figure 2a,b shows the spectra when *x* = 0.05, and the external magnetic field states are 0 and 320 mT, respectively; Figure 2c,d shows when *x* = 0.1, the external magnetic field states are 0 and 320 mT, respectively. The full spectrum analysis of the four experiments was performed using the GSAS software, with the red crosses in the figure representing experimental values, the green line representing fitted values, the black lines at the bottom representing predicted peak positions, and the purple line representing the difference between the experimental and fitted values. The analysis shows that Li_3_V_2_ (PO_4_)_3_/C is a monoclinic crystal with space group *P*1*2*_1_/*c*1, and no impurities are present. In the *x* = 0.1 sample, there is an extra diffraction peak at 7°, which is a signal of the formation of crystalline water by the sample adsorbing water vapor, which can be removed by heating. Comparing the lattice constants *a*, *b*, and *c* in Figure 2a,b, it can be seen that the external magnetic field causes an elongation of the crystal axes, and the same phenomenon also occurs in Figure 2c,d. This indicates that the sample exhibits a magnetostriction.

**Figure 3:**
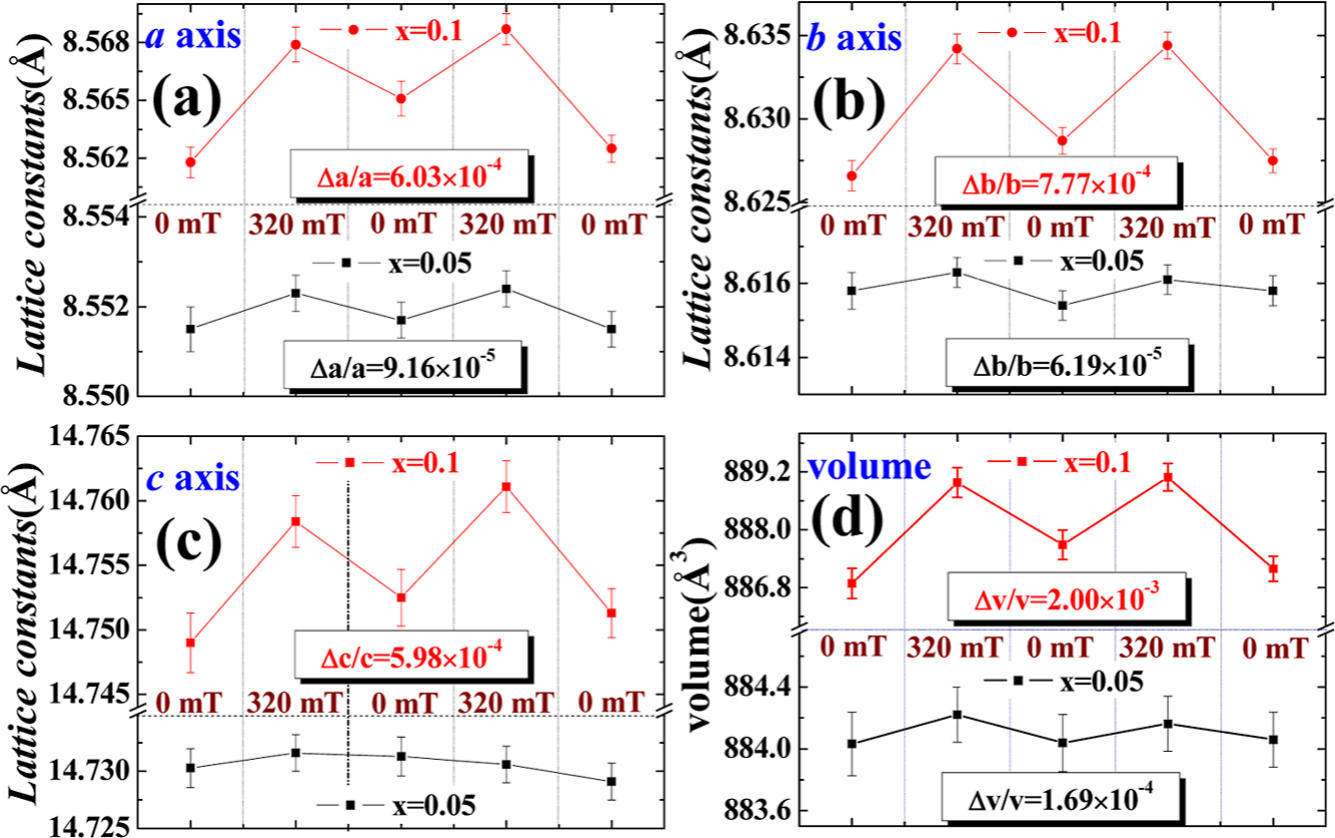
Lattice constants (a) *a*-axis, (b) *b*-axis, (c) *c*-axis, and (d) volume for LVFeP/C with *x* = 0.05 and 0.1 under two cycles of 0–320–0 mT magnetic field.

To confirm the impact of the magnetic field on the crystal structure of the sample, a cycle of zero field—external magnetic field was used to analyze the magnetostriction of the sample. The experiment analyzed the XRD spectra under two cycles of external magnetic field from 0 → 320 → 0 mT. Figure 3a–d shows the resulting plots of the lattice constants and cell volumes against different external magnetic fields. After conducting two cycles of applying an external magnetic field and then demagnetizing it back to zero, no permanent lattice expansion or irreversible contraction was observed. However, magnetically induced expansion did occur, indicating that the ion channels of the material are magnetically controllable.

Under an external magnetic field of 0 and 320 mT, the expansion ratio Δ*a*/*a*, obtained by dividing the expansion amount Δ*a* by the original length of the *a*-axis in Figure 3a, is 9.16 × 10^–5^ for the sample with *x* = 0.05, and 6.03 × 10^–4^ for the sample with *x* = 0.1. This not only indicates that the magnetic field has a significant impact on the *a*-axis but also shows that the magnetic field has a larger effect on the sample with *x* = 0.1. Similarly, in Figure 3b, Δ*b*/*b* = 6.19 × 10^–5^ for *x* = 0.05 and 7.77 × 10^–4^ for *x* = 0.1, showing a greater response of the *b*-axis for the sample with *x* = 0.1. In Figure 3c, it is found that the *c*-axis of the sample with *x* = 0.05 does not show significant changes under the influence of the magnetic field, but the expansion ratio of the *c*-axis of the sample with *x* = 0.1 is 5.98 × 10^–4^, indicating that higher iron ion doping will be subject to a greater effect of the magnetic field to produce the result of magnetic-induced expansion. In Figure 3d, the volume of LiVFeP/C, when *x* = 0.05; the volume expansion ratio Δ*V*/*V* is 1.69 × 10^–4^, when *x* = 0.1, it is 2.00 × 10^–3^. Comparing the same sample, the volume with the applied magnetic field is always larger than that without the magnetic field. The cell volume of the pure LVP sample is 890.1(2) Å^3^, which is larger than the volume of *x* = 0.05 and 0.1. The reason is that the radius of the iron ion is smaller than that of the vanadium ion (Fe^2+^ (0.075 nm) or Fe^3+^ (0.069 nm)), V^3+^ (0.078 nm). When some vanadium ions are replaced, they will lead to a reduction in volume.

The equation *L*_*i*_ = *L*_*i*0_(1 + β_i_Δ*H*) is used to explain the effect of the magnetic field on the crystal axis length. Where *L*_*i*_ is the lattice length in a specific *i* direction, *L*_*i*0_ is the lattice constant under zero magnetic field, β_*i*_ is the magnetostrictive coefficient in the specific *i* direction, and Δ*H* is the change in the magnetic field. Table 2 shows the results obtained. It can be seen that when the iron doping ratio is higher, the lengths of the *a* and *b* axes are more significantly affected by the change in the magnetic field.

**Table 2:** Magnetostriction Coefficients in Different Crystallographic Directions

*x*	β_a_ (mT^–1^)	β_b_ (mT^–1^)	β_c_ (mT^–1^)
0.05	2.87 × 10^–4^	1.94 × 10^–4^	N/A
0.1	1.89 × 10^–3^	2.43 × 10^–3^	1.87 × 10^–3^

**Figure 4:**
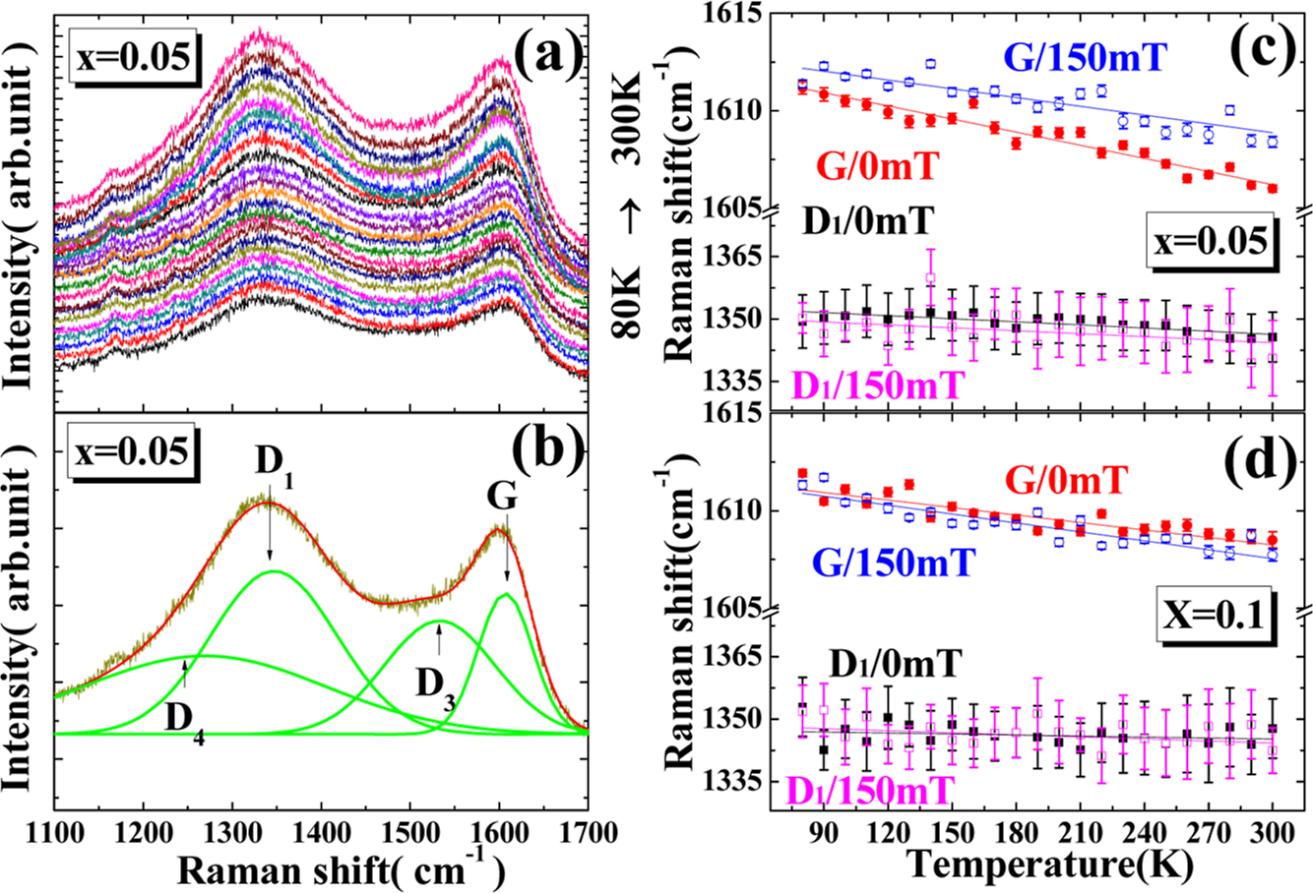
(a) Raman spectra from 80 to 300 K and (b) fitted D_1_, D_3_, D_4_, and G-band for LVFeP/C with *x* = 0.05 under zero magnetic field. Evolution of D_1_ and G-band with temperature for (c) *x* = 0.05 and (d) *x* = 0.1 under *H* = 0 and 150 mT.

Figure 4a displays the Raman spectrum of the *x* = 0.05 sample at zero field from 80 to 300 K. Figure 4b shows a schematic diagram of the Raman spectrum fitting. The corresponding Raman peaks identified in the fitting are labeled as D_4_, D_3_, D_1_, and the G-band. The G-band originates from ordered carbon with sp^2^ hybrid orbitals, while the D-band is attributed to disordered carbon also with sp^2^ hybrid orbitals. Figure 4c,d demonstrates the peak shifts of the D_1_ and G-bands at various temperatures under external magnetic fields of *H* = 0 and 150 mT, for *x* = 0.05 and *x* = 0.1, respectively. Under both *H* = 0 and 150 mT, the shifts in the D_1_-band are not pronounced for *x* = 0.05 and 0.1, but a significant blue shift in the G-band at 150 mT is observed for *x* = 0.05. This shift may be due to the diamagnetic behavior of ordered carbon, which reduces the distance between carbon atoms, thereby increasing their bond strength and causing a blue shift. This suggests that, under the influence of an external magnetic field, the reduction in the spacing of ordered carbon could decrease the size of some ionic channels. Disordered carbon, on the other hand, remains unaffected by the magnetic field. This has significant implications for the material design of future LVP series materials in magnetic environments—specifically, how to reduce the outer layer of ordered carbon and increase disordered carbon to prevent the shrinking of ionic channels due to magnetic effects. Additionally, a temperature increase also leads to a red shift in the G-band, increasing the interatomic distances of ordered carbon. From the changes in the G-band with respect to temperature or the applied magnetic field seen in Figure 4c,d, the equivalent conversion ratio of the external carbon film affected by the magnetic field or temperature can be calculated. When *x* = 0.05, each 1 K rise in temperature is equivalent to a decrease of 1.83 mT in the magnetic field; for *x* = 0.1, each 1 K rise is equivalent to an increase of 4.21 mT in the magnetic field. These results show that an increase in temperature can counteract the magneto-contraction effects of the external magnetic field and increase the size of ionic channels in the region of ordered carbon.

**Figure 5:**
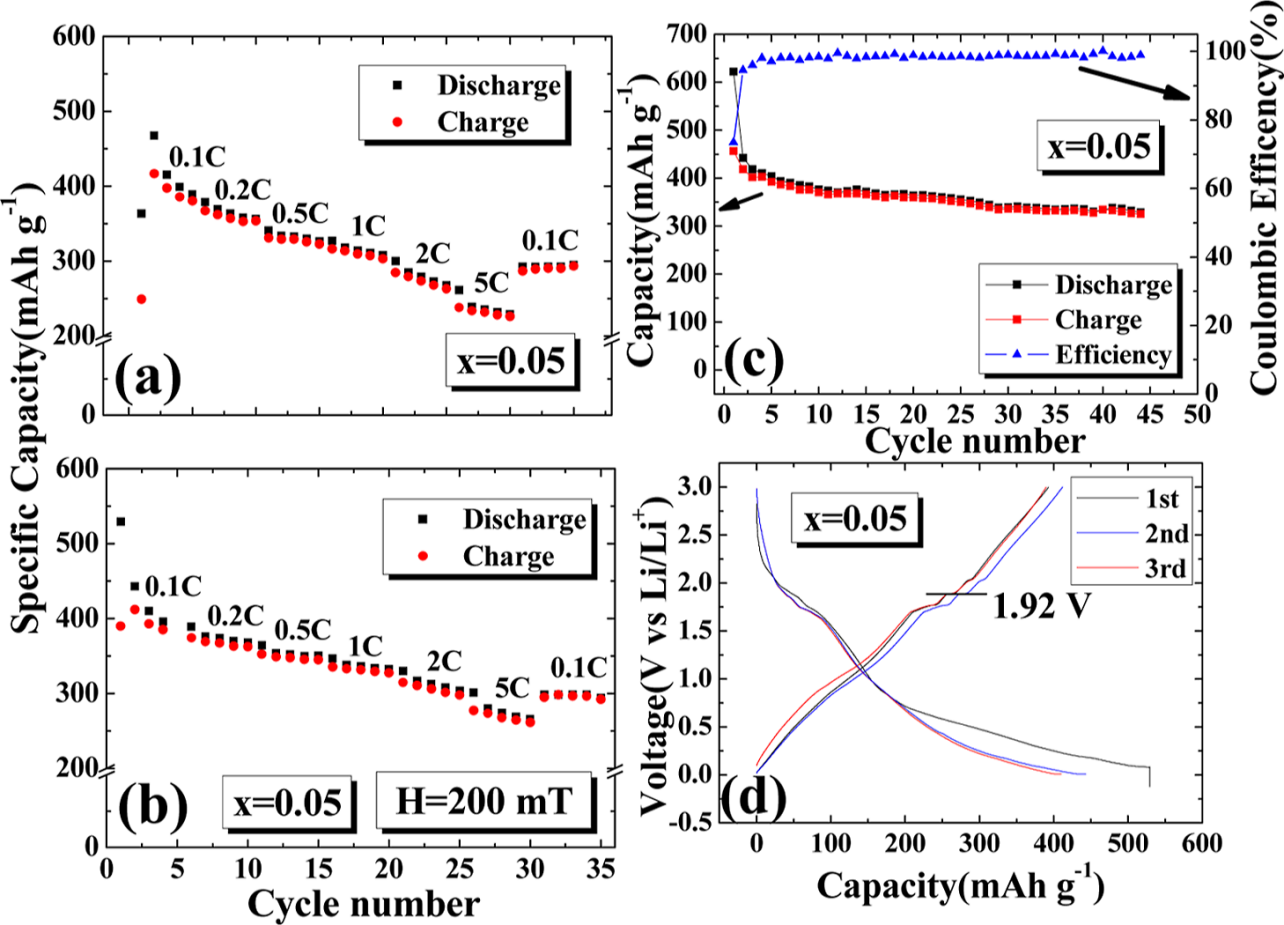
Charge–discharge tests at different C-rates for the *x* = 0.05 sample under an external magnetic field of (a) 0 and (b) 150 mT. Rates range from 0.1, 0.2, 0.5, 1, 2, to 5 C and back to 0.1 C, with each rate tested 5 times in a cycle. (c) Curve and Coulombic efficiency for 45 charge–discharge cycles at 0.1 C. (d) First 3 charge–discharge curves at 0.1 C.

The C-rate is an important parameter for batteries, used to assess the amount of current needed to charge and discharge the battery. One C represents the amount of current that can fully charge the battery in 1 h, while 0.1 C would need 10 h to fully charge the battery. The capacity of the battery that can be used may vary under different charging and discharging currents. The sample with *x* = 0.05 provided a maximum discharge capacity of 467.81 mA h/g at 0.1 C, an average discharge capacity of 417.98 mA h/g, and an average 5 C discharge capacity of 239.45 mA h/g, as shown in Figure 5a. The electric capacity decreases linearly with increasing charging and discharging rates. The variable charging current experiment tested from 0.1, 0.2, 0.5, 1, 2, to 5 C and then back to 0.1 C. When it returned to 0.1 C, the average discharge capacity became 292.88 mA h/g, with a decay rate of approximately 29.93%. This experiment shows that different charging and discharging rates of the battery will cause irreversible capacity loss. Figure 5b shows the variable charging current experiment under the applied magnetic field of 150 mT. The maximum discharge capacity provided by 0.1 C was 529.33 mA h/g, and the average discharge capacity was 416.44 mA h/g. However, compared to the zero-field experiment in Figure 5a, the electric capacity curve falls more gently at different charging and discharging rates, and the average 5 C charging and discharging capacity is 278.07 mA h/g, which is still higher than the zero-field experiment. The average discharge after the variable charging current returns to 0.1 C is 297.53 mA h/g, with a decay rate of 28.55%. The comparison shows better overall performance under the applied magnetic field. In Figure 5c, when *x* = 0.05, the average discharge capacity is 361.16 mA h/g under 0.1 C for 45 cycle charges and discharges, and the Coulomb efficiency is an average of 98.40%, with a decay rate of 19.11% after 45 cycles. Figure 5d shows the first three charge and discharge curves under the charging conditions of *x* = 0.05 and 0.1 C. The charging platform is shown to be at 1.92 V.

**Figure 6:**
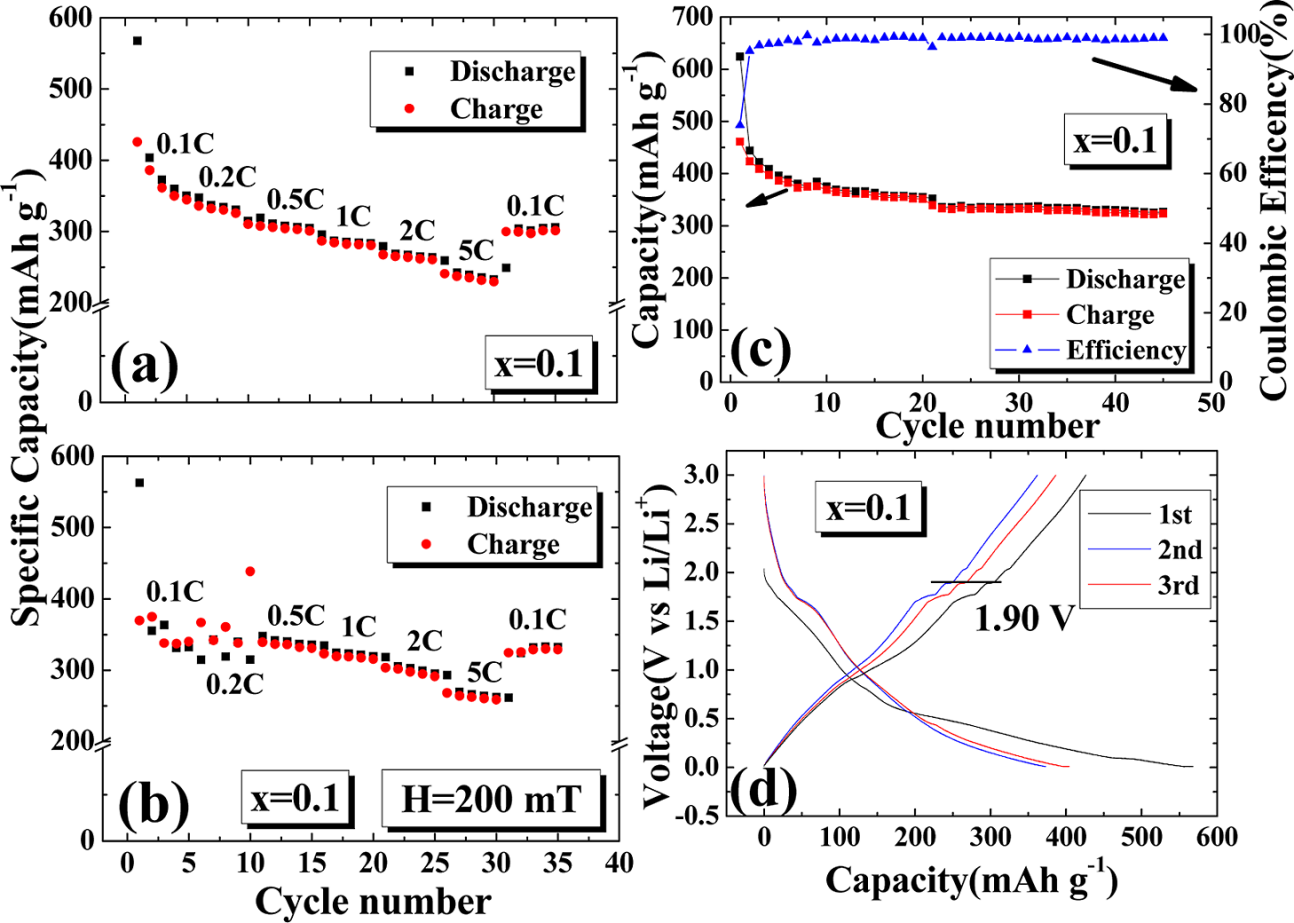
Charge–discharge tests at different C-rates for the *x* = 0.1 sample under an external magnetic field of (a) 0 and (b) 150 mT. Rates range from 0.1, 0.2, 0.5, 1, 2, to 5 C and back to 0.1 C, with each rate tested 5 times in a cycle. (c) Capacity and Coulombic efficiency changes during 45 charge–discharge cycles. (d) Capacity and voltage relationship for the first three charge–discharge cycles.

Figure 6a shows that when *x* = 0.1, the maximum discharge capacity at 0.1 C can reach 567.86 mA h/g, the average discharge capacity is 371.83 mAh/g, and the average 5 C discharge capacity is 241.98 mA h/g. In the variable charging current experiment, the capacity also decreases linearly, and from the second 0.1 C, the average discharge capacity of the battery is 293.18 mA h/g with a decay rate of 21.15%. This also shows that after different rates, the battery capacity will be consumed, and the capacity is irreversible. Figure 6b shows the charging and discharging experiments with an added magnetic field, which provides a maximum discharge capacity of 563.11 mA h/g at 0.1 C, an average discharge capacity of 345.59 mA h/g, and an average 5 C charging and discharging capacity of 271.07 mA h/g, which is higher than that in the zero magnetic field. The average discharge amount returned to 0.1 C after the variable charging current experiment is 316.64 mA h/g, with a decay rate of 8.37%. The experiment with an added magnetic field showed that the battery has a lower decay rate and a better fast charging performance. Figure 6c shows that when *x* = 0.05, under 0.1 C, after 45 cycles of charging and discharging, the average discharge capacity is 353 mA h/g, the Coulomb efficiency is on average 98.57%, and the decay rate after 45 times is 23.27%. Figure 6d shows the first three charging and discharging curves under the charging conditions of *x* = 0.1 and 0.1 C. The charging platform at 1.90 V is shown in the process.

Comparing *x* = 0.05 and *x* = 0.1, when slow charging and discharging at 0.1 C, *x* = 0.05 has better average capacity performance under zero magnetic field, but the *x* = 0.1 sample has a lower decay rate. Both samples will be interfered with by the added magnetic field, but the *x* = 0.1 sample can better resist the changes caused by the added magnetic field.

When the batteries were subjected to C-rate testing, the sample with *x* = 0.05 showed a capacity decay rate of approximately 29.93% under 0 mT, and a decay rate of 28.55% under a 200 mT magnetic field. Therefore, the magnetic field did not significantly enhance or suppress the aging degradation effect for the *x* = 0.05 sample. On the other hand, the sample with *x* = 0.1 had a capacity decay rate of 21.15% under a zero magnetic field, which significantly decreased to 8.37% under a 200 mT magnetic field. Comparatively, the sample doped with 10% Fe ions showed better antiaging effects than the 5% doped sample under zero magnetic field.

On a microscopic level, this 7.4% (29.93–21.15% = 8.78%) improvement could stem from two aspects: the additional 5% Fe ion doping and the relatively significant increases in *R*_e_, *R*_SEI_, and *R*_Ct_. However, under an applied magnetic field, the decay rate of the *x* = 0.1 sample decreased by 20.18% (28.55–8.37%) compared to the *x* = 0.05 sample. Given that the values of *R*_e_, *R*_SEI_, *R*_Ct_, and the diffusion coefficient *D* are similar to those under zero magnetic field, the prolonged reduction in the aging effect from multiple charge–discharge cycles is more likely due to the greater magnetostrictive expansion caused by the higher Fe ion doping.

**Figure 7:**
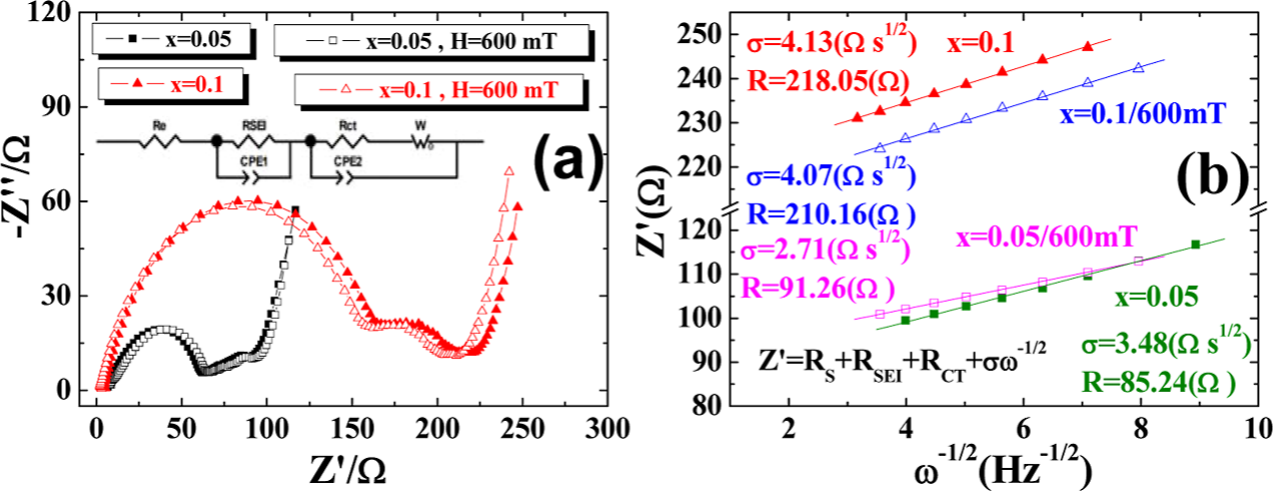
*x* = 0.05 and *x* = 0.1, with and without a magnetic field. (a) EIS spectra and (b) diffusion rate linear analysis.

EIS uses a 5 mV alternating current voltage to scan the impedance at different frequencies from 10^–2^ to 10^5^ Hz. In Figure 7a, Re represents the resistance of the electrolyte; *R*_SEI_ represents the resistance of the solid electrolyte interface film; *R*_Ct_ is the charge transfer resistance; CPE is the double-layer capacitance of the electrode surface; and *W* is Warburg impedance. The high-frequency area in the figure is the left semicircle, which is mainly related to the charge transfer resistance of the electrolyte and lithium metal; the mid-frequency area is the small semicircle in the middle, which is mainly related to the charge transfer resistance of the electrolyte and LVFeP/C; and the low-frequency area is the right line, mainly representing lithium–ion diffusion in the electrode. Figure 7b shows the linear regression of the Warburg impedance curve to obtain σ and *R* values. Table 3 uses the PS Trace 5.8 program to fit the resistance values in the table from Figure 7a. It can be seen that the *R*_Ct_ resistance value of *x* = 0.1 is nearly 2.32 times larger than that of the *x* = 0.05 sample. The literature points out that the high and low *R*_Ct_ will affect the kinetics of the battery, so *x* = 0.05 is more conducive to the electrochemical performance of lithium–ion insertion and migration compared to *x* = 0.1. The diffusion coefficient *D* formula^37^ is
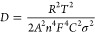
1awhere *R* is the gas constant (*R* = 8.314 J mol^–1^ K^–1^), *T* is the temperature (*T* = 300 K), *A* is the area of the electrode surface (*A* = 1.54 cm^2^), *n* is the number of electrons per molecule during oxidation (*n* = 2), *F* is Faraday’s constant (*F* = 96486 C mol^–1^), σ is the Warburg factor obtained from eq 2, ω is angle frequency, and *C* is the concentration of lithium ions, calculated using eq 3
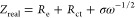
2a

**Table 3:** Resistance Values and Diffusion Rates in the EIS for *x* = 0.05 and *x* = 0.1, with and without a Magnetic Field

Li_3_(V_1–*x*_Fe_*x*_)_2_ (PO_4_)_3_/C				
*x*	0.05		0.1	
*H*	0 mT	600 mT	0 mT	600 mT
*R*_e_ (Ω)	6.12	7.60	4.44	2.09
*R*_SEI_ (Ω)	2.16	3.87	39.81	35.21
*R*_Ct_ (Ω)	56.48	56.12	130.21	131.00
*D* (10^–13^ m^2^ s^–1^)	3.49	5.77	2.50	2.58

The concentration of lithium ions (*C*) was determined by
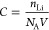
3where *n*_Li_ represents the number of Li^+^ ions in each LVFeP unit cell (*n*_Li_ = 8), *N*_A_ is Avogadro’s constant (*N*_A_ = 6.02 × 10^23^ mol^–1^), and *V* is the unit cell volume of LVFeP obtained from structural refinement, as shown in Figure 3d (886.8 for *x* = 0.1 and 884.0 Å^3^ for *x* = 0.05). All of the required fitting parameters are listed in Table 3.

Table 3 also shows that the sample with *x* = 0.05 has a higher diffusion coefficient than the *x* = 0.1 sample. Under a 600 mT magnetic field, the diffusion coefficients of both samples experience a slight increase, suggesting that the external magnetic field may slightly enhance ion diffusion, although the effect is not very pronounced. This implies that the setup of an external magnetic field parallel to the electrodes does not significantly affect the path of ions from leaving the carbon layer to the lithium metal electrode. Additionally, as indicated by the *R*_SEI_ and *R*_Ct_ values in Table 3, the *x* = 0.05 sample has a lower total internal resistance, thus exhibiting better intrinsic battery parameters regardless of the presence of an external magnetic field.

**Figure 8:**
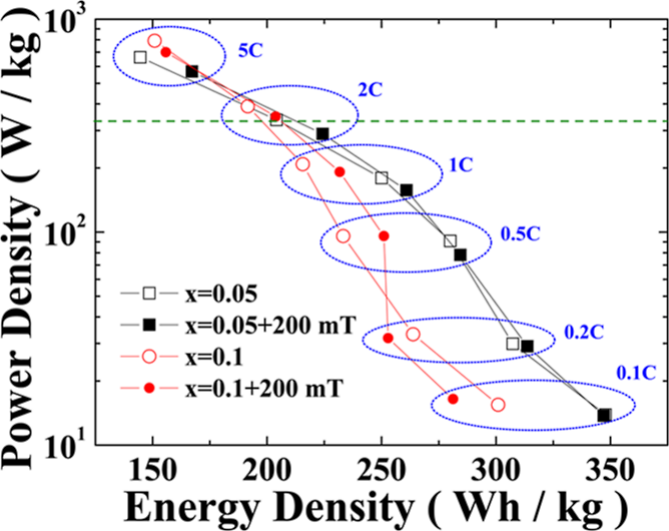
Energy density versus power density plots for *x* = 0.05 and *x* = 0.1, under zero external magnetic field and an applied 200 mT magnetic field.

Figure 8 displays the energy versus power density plots for *x* = 0.05 and *x* = 0.1 under zero external magnetic field (hollow symbols) and an applied 200 mT magnetic field (solid symbols), where each data point represents the average of five experiments conducted under identical conditions. The figure shows that, for the *x* = 0.05 sample, an external magnetic field not only slightly reduces the power density but also slightly increases the energy density. For the *x* = 0.1 sample, the same trend is observed at discharge rates above 0.5 C, but at C rates less than 0.5 C, the energy density under an applied magnetic field is lower than that in the zero magnetic field tests. Overall, for the *x* = 0.05 sample with low iron doping, the impact of the external magnetic field is less pronounced; for the *x* = 0.1 sample with higher iron doping, the effect of the external magnetic field on energy versus power density is related to the discharge rate. Furthermore, regardless of the presence of an external magnetic field, the energy versus power density performance of the *x* = 0.05 sample is superior to that of the *x* = 0.1 sample. The performance of *x* = 0.05 and *x* = 0.1 on the energy versus power density plots may be attributed to the result of several combined effects. Under an external magnetic field, both samples exhibit magnetostrictive expansion, with the sample doped with a higher concentration of iron, demonstrating a greater expansion coefficient. For the *x* = 0.05 sample, the outer ordered carbon layer undergoes magnetostrictive contraction due to the magnetic field. The effect of the magnetic field on the rate of ion diffusion from the LVFeP carbon layer to the lithium metal electrode is not pronounced. Consequently, the overall energy versus power density performance of the *x* = 0.05 sample is less impacted by the magnetic field. The primary reason might be due to the expansion effect of the magnetic field on LVFeP being offset by the contraction of the outer ordered carbon layer; meanwhile, the outer ordered carbon layer of the *x* = 0.1 sample is not significantly affected by the magnetic field. Thus, under an external magnetic field, the magnetostrictive expansion of LVFeP opens up internal channels, leading to notable changes in battery performance. This allows for a higher release of ions per unit time at discharge rates above 0.5 C, exhibiting an increased energy density. At discharge rates below 0.5 C, as fewer ions are released per unit time, the effect of the size of the internal channels is less evident. However, since the *x* = 0.1 sample contains a higher quantity of iron ions, which may experience greater orbital and spin state effects under a magnetic field, leading to electrochemical valence shifts that affect lithium ion release,^38^ it displays a lower energy density.

## 4 Conclusions

This research demonstrates that doping 5 and 10% magnetic iron atoms at the vanadium sites in LVP/C results in the formation of Li_3_(V_1–*x*_Fe_*x*_)_2_(PO_4_)3/C materials. Both maintain the same monoclinic crystal structure as LVP, belonging to the *P*1*2*_1_/*c*1 space group, and exhibit magnetostriction effects. Raman spectroscopy shows that, for the *x* = 0.05 sample, the outer ordered carbon layer exhibits magnetostrictive contraction, whereas the disordered carbon in both samples and the ordered carbon in the *x* = 0.1 sample do not show significant Raman peak shifts in relation to the applied magnetic field. C-Rate and cyclic charge–discharge tests indicate that under a zero magnetic field, the *x* = 0.05 sample exhibits superior average capacity performance, while the *x* = 0.1 sample demonstrates a lower decay rate. Both samples exhibit an influence from the magnetic field, particularly in the study of aging effects. In the C-rate tests under an applied magnetic field, the sample with *x* = 0.1 showed a relative reduction in decay rate by 20.18% compared to the sample with *x* = 0.05. This may be related to the additional 5% Fe ion doping. EIS experiments show slight improvements in ion diffusion rates for both samples, although the effects are not pronounced. Regardless of the presence of an external magnetic field, the *x* = 0.05 sample exhibits lower total internal resistance, thus demonstrating better intrinsic battery parameters. By examining the performance in terms of energy and power density, the suitable applications for the *x* = 0.05 and *x* = 0.1 samples can be discerned. The *x* = 0.05 sample is suitable for scenarios requiring higher energy density and is less sensitive to magnetic fields, whereas the *x* = 0.1 sample is suitable for applications requiring higher power density output, particularly under high C-rate conditions, and can utilize magnetic fields to adjust its energy density and cycling performance.
